# Mechanistic Insights into Autophagy-Dependent Cell Death (ADCD): A Novel Avenue for Cancer Therapy

**DOI:** 10.3390/cells14141072

**Published:** 2025-07-13

**Authors:** Md Ataur Rahman, Maroua Jalouli, Mohammed Al-Zharani, Ehsanul Hoque Apu, Abdel Halim Harrath

**Affiliations:** 1 Department of Oncology, Karmanos Cancer Institute, Wayne State University, Detroit, MI 48201, USA; 2 Department of Biology, College of Science, Imam Mohammad Ibn Saud Islamic University (IMSIU), Riyadh 11623, Saudi Arabia; mejalouli@imamu.edu.sa (M.J.); mmylzahrani@imamu.edu.sa (M.A.-Z.); 3 Department of Biomedical Sciences, College of Dental Medicine, Lincoln Memorial University, Knoxville, TN 37923, USA; ehsanul.hoqueapu@lmunet.edu; 4 Zoology Department, College of Science, King Saud University, P.O. Box 2455, Riyadh 11451, Saudi Arabia

**Keywords:** autophagy, autophagy-dependent cell death, cancer therapy, molecular mechanisms, tumor microenvironment

## Abstract

Autophagy-dependent cell death (ADCD) presents a promising but challenging therapeutic strategy in cancer treatment. Autophagy regulates cellular breakdown and stress responses, serving a dual function—either inhibiting tumorigenesis or facilitating the survival of cancer cells in advanced stages. This paradox presents both opportunities and challenges in the exploration of autophagy as a potential target for cancer treatment. In this review, we explore various pharmacological agents, including autophagy inhibitors (e.g., chloroquine, 3-MA) and activators (e.g., rapamycin, metformin), which have demonstrated effectiveness in modulating autophagy-dependent cell death (ADCD). These agents either enhance cancer cell apoptosis or sensitize tumors to conventional therapies. Combination therapies, such as the use of autophagy modulators alongside chemotherapy, immunotherapy, or radiation therapy, offer enhanced therapeutic potential by overcoming drug resistance and improving overall treatment efficacy. Nonetheless, significant challenges remain, including tumor heterogeneity, treatment resistance, and off-target effects of autophagy-targeting agents. Future progress in biomarker discovery, precision medicine, and targeted medication development will be crucial for enhancing ADCD-based methods. Although autophagy-dependent cell death presents significant potential in cancer treatment, additional studies and clinical validation are necessary to confirm its position as a conventional therapeutic approach. Therefore, this review aims to identify the existing restrictions that will facilitate the development of more effective and personalized cancer therapies, hence enhancing patient survival and outcomes.

## 1. Introduction

Cancer remains a significant worldwide health problem, responsible for millions of deaths annually, despite continuous progress in early identification, diagnostics, and treatment [1]. Traditional therapeutic methods, including chemotherapy, radiation, and immunotherapy, have markedly enhanced patient outcomes [2]. Nonetheless, treatment resistance, tumor heterogeneity, and severe side effects persist in constraining the efficacy of these strategies [3]. Therefore, there is an immediate necessity to investigate alternative therapeutic targets and innovative methods of cell death that might be utilized for cancer treatment.

Autophagy-dependent cell death (ADCD) is a form of regulated cell death (RCD) that utilizes the autophagy mechanism explicitly to facilitate cellular destruction. Unlike other programmed cell death mechanisms, such as apoptosis or necroptosis, where autophagy may be involved but is not solely responsible, ADCD relies on an efficient autophagy system for its successful execution [4]. In contrast to apoptosis, which is extensively described and commonly targeted in cancer treatment, ADCD is comparatively under-researched. Nonetheless, increasing evidence suggests that excessive or dysregulated autophagy may lead to cellular death, providing a potential method for eliminating cancer cells [5]. Autophagy, a catabolic mechanism that degrades and recycles cellular components, is crucial for sustaining cellular homeostasis [4,6]. It enables cells to endure adverse circumstances, including nutritional scarcity, oxidative stress, and hypoxia, by destroying impaired organelles and misfolded proteins [6]. In cancer, autophagy plays a dual role, functioning as a tumor suppressor in early-stage malignancies and as a tumor promoter in advanced tumors, facilitating cancer cell survival under metabolic and pharmacological stress [7]. The contradictory function of autophagy in cancer advancement emphasizes the intricacy of this phenomenon and stresses the necessity for a more profound comprehension of its molecular underpinnings.

Autophagy is meticulously regulated by a network of signaling pathways and autophagy-related genes (ATGs), which control its onset, advancement, and termination [8]. The process encompasses multiple essential stages: initiation, nucleation, elongation, and destruction, facilitated by a fundamental group of regulatory proteins [9]. Beclin-1 is a pivotal regulator of autophagy, forming a complex with class III phosphatidylinositol 3-kinase (PI3K) to commence autophagosome production [10]. The transformation of cytosolic LC3-I into membrane-associated LC3-II signifies the maturity of autophagosomes, enabling their fusion with lysosomes for the degradation and recycling of cellular constituents [11]. Although autophagy generally functions as a survival strategy, excessive autophagic activity can induce cell death, particularly when it leads to the indiscriminate destruction of vital cellular components.

The regulation of ADCD is influenced by numerous intracellular and extracellular factors, including metabolic stress, oxidative damage, and interactions with alternative cell death pathways [12]. The interaction among autophagy, apoptosis, and necroptosis is notably critical in cancer [13], as these pathways share regulatory factors and can influence one another's activation. Proteins, including p53, Bcl-2, and caspases, are recognized for their role in modulating both apoptotic and autophagic cell death, thereby introducing further complexity to the regulation of cancer cell fate [14]. Autophagy may serve as a precursor to apoptosis, facilitating cell survival under moderate stress while inducing cell death under extreme stress [15]. In certain instances, autophagy may function as an autonomous cell death mechanism, distinct from apoptosis and necroptosis, providing an alternative method for eradicating cancer cells that are resistant to conventional treatments [16].

Therefore, ADCD is an innovative and promising strategy for cancer treatment. The dual role of autophagy in cancer complicates therapeutic targeting; nonetheless, advancements in understanding its molecular underpinnings have facilitated the development of novel therapy regimens [17]. By specifically regulating autophagy, it may be possible to utilize this mechanism to enhance cancer cell mortality, overcome drug resistance, and improve the efficacy of current treatments. This review would like to suggest that autophagy-based therapeutic techniques could transform cancer treatment and offer renewed hope for patients with drug-resistant and aggressive malignancies.

## 2. Molecular Mechanisms of Autophagy and Autophagy-Dependent Cell Death

**Figure 1:**
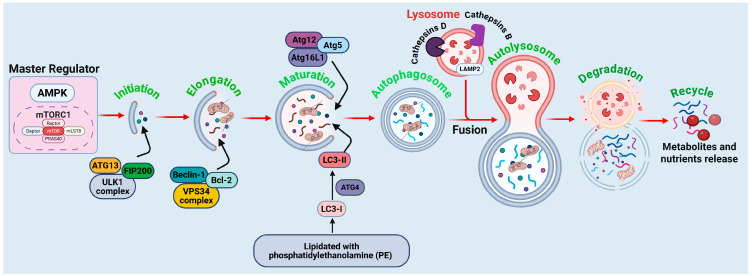
Molecular mechanism of autophagy. The initiation phase is governed by the ULK1 complex (comprising ULK1, ATG13, and FIP200), which is activated by AMPK and suppressed by mTORC1 signaling per food availability. Throughout elongation and maturation, the Beclin-1–VPS34 complex initiates the phagophore, whilst the ATG12-ATG5-ATG16L1 complex and lipidated LC3-II facilitate membrane extension and cargo encapsulation. Bcl-2 exerts an adverse regulatory effect on Beclin-1, hence inhibiting autophagy. During the autophagosome development phase, mature autophagosomes enclose cytoplasmic constituents, such as impaired organelles and proteins. The fusion step involves the docking of lysosomes through LAMP2, resulting in the formation of autolysosomes, where lysosomal enzymes, such as cathepsins B and D, destroy the cargo. The degradation and recycling phase facilitates the release of amino acids, lipids, and other metabolites, thus reinstating cellular equilibrium and aiding metabolic adaptability. The figure is original and was created and generated by BioRender.com, an online commercial platform.

Considering the regulatory pathways of autophagy offers significant insights into potential therapeutic strategies for cancer treatment, especially in utilizing autophagy-dependent cell death as an innovative anticancer method. The molecular mechanisms of autophagy are illustrated in Figure 1, with detailed descriptions provided in the following sections.

### 2.1. Autophagy and Its Regulatory Pathways

Autophagy is a precisely regulated degradation mechanism that removes damaged organelles, misfolded proteins, and pathogens [18]. The process is facilitated by a collection of autophagy-related genes (ATGs) and can be generally classified into the following stages.

#### 2.1.1. Initiation of Autophagy

The initial phase of autophagy is precisely controlled by the Unc-51-like kinase 1 (ULK1) complex, which functions as a crucial indicator of cellular stress [5]. Under typical circumstances, the mechanistic target of rapamycin complex 1 (mTORC1) suppresses ULK1 activity by phosphorylating ATG13 and FIP200, thereby preventing the initial phase of autophagy [19]. Under conditions such as nutritional deprivation or metabolic stress, AMP-activated protein kinase (AMPK) suppresses mTORC1 and directly activates ULK1 via phosphorylation [20]. The activated ULK1 complex, comprising ULK1, ATG13, and FIP200, subsequently phosphorylates downstream targets, facilitating the recruitment of other autophagy-related proteins essential for autophagosome formation [21]. This initial stage is crucial for determining whether autophagy serves as a survival or cell death mechanism in cancer cells.

#### 2.1.2. Nucleation: Formation of the Phagophore

During the initiation of autophagy, the subsequent phase involves nucleation, which is governed by the Beclin-1 (ATG6)-Vps34 complex. Beclin-1, a tumor suppressor protein, forms a core complex with class III phosphatidylinositol 3-kinase (PI3K), also known as Vps34 [7]. This complex is crucial for synthesizing phosphatidylinositol-3-phosphate (PI3P), a lipid that attracts supplementary autophagy-related proteins required for phagophore formation. The activity of the Beclin-1 complex is stringently regulated by interactions with regulatory proteins, including Bcl-2 and Bcl-xL, which can obstruct autophagy by sequestering Beclin-1 [22]. In contrast, pro-autophagic signals, such as BH3-only proteins (e.g., Bad, Bnip3), can interfere with the Beclin-1-Bcl-2 connection, thereby facilitating autophagy [23]. The nucleation phase is pivotal in ascertaining whether autophagy promotes cell survival or advances to autophagy-mediated cell death.

#### 2.1.3. Elongation and Maturation of Autophagosomes

After nucleation, the phagophore extends to create a double-membraned autophagosome that encases cytoplasmic material. This process is facilitated by two ubiquitin-like conjugation systems: the ATG12-ATG5-ATG16L1 complex and the LC3 (microtubule-associated protein 1A/1B-light chain 3) conjugation system [24]. In the initial system, ATG12 is covalently linked to ATG5 in a manner reliant on ATG7 and ATG10 [25]. The ATG12-ATG5 complex subsequently engages with ATG16L1, creating a scaffold essential for the elongation of the autophagosome membrane [26]. In the second conjugation method, LC3 is subjected to proteolytic cleavage by ATG4, resulting in the formation of LC3-I, which is subsequently lipidated with phosphatidylethanolamine (PE) to produce LC3-II [27]. LC3-II is a crucial indicator of autophagosome formation, playing a role in cargo selection and membrane expansion [28]. LC3-II on autophagosomes promotes their fusion with lysosomes, indicating the onset of the degradation phase.

#### 2.1.4. Fusion with Lysosomes and Cargo Degradation

The finalizing phase of autophagy entails the fusion of autophagosomes with lysosomes, resulting in the formation of autolysosomes, where the enclosed cellular components undergo degradation and recycling [29]. The mediation of this process involves SNARE proteins, particularly syntaxin-17 (STX17), alongside small GTPases like Rab7 [30]. Cathepsin D, a lysosomal enzyme, degrades autolysosomal contents, facilitating the release of amino acids, fatty acids, and other macromolecules into the cytoplasm for reutilization [31]. The effective execution of this step influences the outcome of autophagic activity. Controlled autophagic degradation supports cellular survival by maintaining metabolic balance. Excessive autophagy may lead to autophagy-dependent cell death, particularly in cancer cells, through the depletion of essential cellular components and the initiation of a metabolic catastrophe.

### 2.2. Molecular Mechanisms of Autophagy-Dependent Cell Death

**Figure 2:**
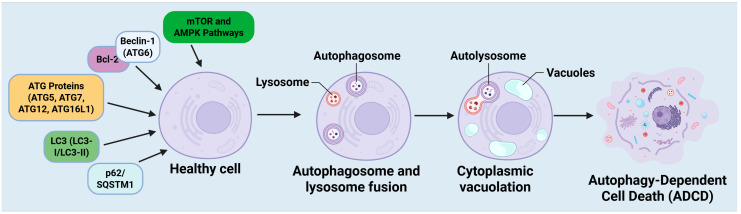
Mechanism of autophagy-dependent cell death (ADCD). Initiation of ADCD from a healthy cell. Essential regulatory molecules, such as Beclin-1 (ATG6), ATG proteins (ATG5, ATG7, ATG12, ATG16L1), LC3 (LC3-I/LC3-II), p62/SQSTM1, and the upstream regulators mTOR and AMPK pathways, coordinate the onset and advancement of autophagy. In reaction to cellular stress, autophagosomes are formed and subsequently merge with lysosomes to create autolysosomes. Continuous autophagic activity causes cytoplasmic vacuolation and the breakdown of vital cellular components, ultimately culminating in ADCD. The function of excessive or dysregulated autophagy in facilitating non-apoptotic, planned cell death marked by significant vacuolization and organelle degradation. The figure was modified and created using the BioRender.com online commercial platform.

Autophagy-dependent cell death (ADCD) is a regulated form of cell death characterized by excessive or dysregulated autophagy, which results in the degradation of vital cellular components and ultimately leads to cell death [32]. Multiple essential molecular components govern this process, encompassing autophagy-related proteins (ATGs), signaling pathways, and specific autophagic adaptors. The interaction among these elements dictates whether autophagy serves as a protective mechanism or facilitates cell death in cancer (Figure 2).

#### 2.2.1. Beclin-1 (ATG6): A Principal Regulator of Autophagy

Beclin-1 (ATG6) is a fundamental regulator of autophagy and is essential for commencing the process [33]. It constitutes the fundamental element of the class III phosphatidylinositol 3-kinase (PI3K) complex, which is crucial for the nucleation of autophagosomes. Beclin-1 engages with many regulatory proteins, such as Bcl-2, Bcl-xL, and AMBRA1, to regulate autophagy [34]. In cancer, Beclin-1 is often dysregulated, exhibiting loss-of-function mutations or diminished expression in several malignancies, including breast, ovarian, and prostate cancers [35]. The downregulation of Beclin-1 hinders autophagy, allowing cancer cells to evade autophagic cell death and facilitating tumor progression [36]. In contrast, the overexpression of Beclin-1 can stimulate autophagy and augment ADCD, positioning it as a potential therapeutic target for cancer treatment [36]. The equilibrium between Beclin-1-mediated autophagy and apoptosis is crucial in determining cell fate, particularly in response to chemotherapeutic agents that induce autophagy-dependent cytotoxicity.

#### 2.2.2. ATG Proteins: Crucial for Autophagosome Formation

Autophagy-related (ATG) proteins are essential for the development and maturation of autophagosomes. ATG5, ATG7, ATG12, and ATG16L1 are pivotal in the processes of autophagic membrane elongation and autophagosome maturation [37]. ATG5 is essential for the initiation of autophagy and the creation of autophagosomes [38]. It creates a conjugate complex with ATG12 and ATG16L1, promoting membrane expansion. Research indicates that ATG5 can interact with apoptotic pathways, as cleaved ATG5 translocates to the mitochondria, facilitating the release of cytochrome and apoptosis [39]. ATG5's dual function in autophagy and apoptosis highlights its significance in determining whether autophagy leads to cell survival or autophagic cell death [40]. ATG7 operates as an E1-like enzyme essential for the lipidation of LC3, a critical process in autophagosome maturation [41]. The absence of ATG7 hinders autophagy and exacerbates metabolic stress, potentially affecting tumor advancement [42]. Conversely, persistent ATG7 expression in certain cancer types has been linked to autophagy-induced cell death, suggesting its potential as a therapeutic target.

#### 2.2.3. LC3: An Indicator of Autophagosome Formation

Microtubule-associated protein 1A/1B-light chain 3 (LC3) is a well-established indicator of autophagosome development [43]. LC3 appears in two forms: LC3-I (cytosolic) and LC3-II (membrane-bound), with LC3-II integrated into autophagosomal membranes [44]. The transformation of LC3-I to LC3-II is crucial for the maturation of autophagosomes and the breakdown of cargo [45]. Within the framework of ADCD, LC3 plays a pivotal role in selective autophagy, facilitating the degradation of impaired organelles and protein aggregates. Elevated LC3-II levels are linked to augmented autophagic flux, perhaps facilitating cell death in response to chemotherapeutic drugs [46]. LC3 additionally engages with specific autophagy adaptors, such as p62/SQSTM1, thereby modulating the equilibrium between cellular survival and autophagic cell death [47].

#### 2.2.4. p62/SQSTM1: A Mediator of Autophagy and Apoptosis Interference

p62/SQSTM1 is a specific autophagy receptor that promotes the degradation of ubiquitinated proteins through its interaction with LC3-II [8]. It serves as a conduit between autophagy and apoptosis, affecting cellular destiny under stress conditions. Under typical circumstances, p62 increases when autophagy is compromised, resulting in the accumulation of protein aggregates and oxidative damage [48]. In the context of ADCD, excessive autophagic degradation of p62 may induce apoptosis, as p62 plays a crucial role in regulating the nuclear factor-κB (NF-κB) pathway, which is essential for cell survival [49]. In cancer, p62 exhibits a dual function, acting as a tumor suppressor in certain settings and facilitating carcinogenesis in others [50]. Targeting p62-mediated autophagy regulation has become a promising approach for enhancing the sensitivity of cancer cells to therapy-induced ADCD.

#### 2.2.5. mTOR and AMPK Signaling: Principal Regulators of Autophagy

Autophagy is regulated by two principal signaling pathways: the mammalian target of rapamycin (mTOR) pathway and the AMP-activated protein kinase (AMPK) pathway [51]. The mTOR pathway serves as a principal negative regulator of autophagy, suppressing ULK1 activity in nutrient-abundant environments [52]. In the presence of enough nutrition, mTORC1 phosphorylates ULK1, hence inhibiting the beginning of autophagy [53]. In cancer, mTOR hyperactivation inhibits autophagy, hence enhancing tumor cell viability [54]. Pharmacological inhibition of mTOR with rapamycin or other mTOR inhibitors can elicit ADCD, rendering it a viable therapeutic strategy [46]. The AMPK pathway functions as a metabolic sensor, initiating autophagy in response to energy deficiency. Following ATP depletion, AMPK phosphorylates ULK1 and inhibits mTORC1, thereby initiating autophagy by ADCD [55]. In cancer, AMPK activation can either enhance cell survival or trigger ADCD, contingent upon the cellular context and metabolic condition [56]. Rapamycin induces autophagy in osteosarcoma cells (SaOS-2, U-2OS), triggering ADCD [57]. Targeting AMPK to regulate autophagy is being investigated as a method to increase cancer cell vulnerability to therapy-induced autophagic death.

### 2.3. Crosstalk Between Autophagy-Dependent Cell Death and Other Cell Death Pathways

**Figure 3:**
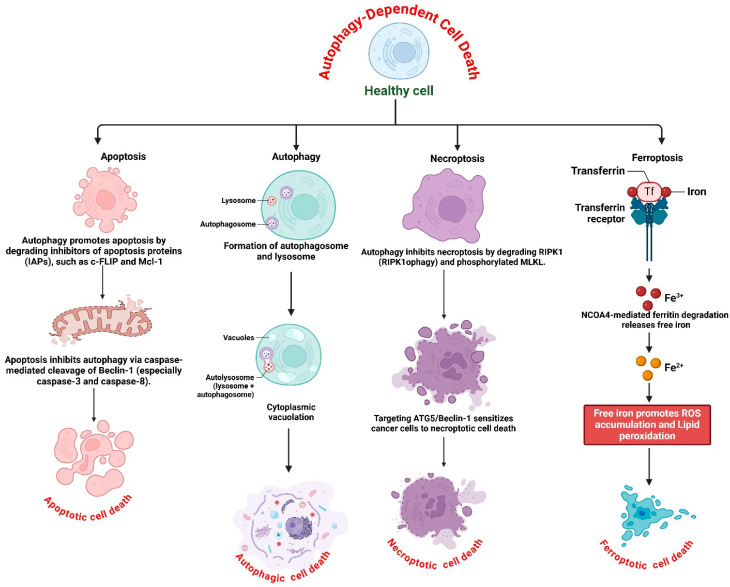
Interplay between autophagy-dependent cell death and other pathways. Modulating autophagy may serve as a potential therapeutic strategy in cancer treatment by influencing apoptosis, necroptosis, and ferroptosis. Autophagy modulates apoptosis by degrading anti-apoptotic proteins, including Mcl-1 and c-FLIP, hence enhancing apoptotic sensitivity. Conversely, apoptosis inhibits autophagy by caspase-3/8-mediated cleavage of Beclin-1, therefore favoring apoptotic cell death. Chemotherapeutic agents, such as doxorubicin and cisplatin, promote autophagy, thereby enhancing apoptosis in cancer treatment. Autophagy suppresses necroptosis by modulating RIPK1 (RIPK1ophagy) and phosphorylated MLKL, hence obstructing necroptotic execution. Autophagy inhibition leads to the accumulation of RIPK1, triggering necroptotic cell death. Targeting autophagy-related genes, such as ATG5 or Beclin-1, enhances the susceptibility of tumor cells to necroptosis. Ferritinophagy, facilitated by NCOA4, induces ferroptosis by liberating iron, enhancing ROS generation, and promoting lipid peroxidation. Inhibiting autophagy with chloroquine or bafilomycin A1 obstructs ferritinophagy, thereby averting ferroptosis. Conversely, ferroptosis inducers like erastin and RSL3 promote ferritinophagy, hence intensifying ferroptotic cell death. The figure was modified and created using the BioRender.com online commercial platform.

The interaction between ADCD and apoptosis, necroptosis, and ferroptosis influences cellular fate in various physiological and pathological contexts, including cancer. Understanding this interaction is crucial for developing modified therapeutic strategies that utilize autophagy modulation to enhance cancer treatment. The complex relationships between ADCD and three principal cell death pathways: apoptosis, necroptosis, and ferroptosis are presented in Figure 3.

#### 2.3.1. Autophagy and Apoptosis: A Dual Regulatory Mechanism

Apoptosis is a defined type of programmed cell death regulated by caspase activation, marked by DNA fragmentation, chromatin condensation, and membrane blebbing [58]. Autophagy and apoptosis are frequently interrelated, as autophagy may operate independently or modulate apoptosis via several molecular processes [59]. A critical element of this interaction is the degradation of anti-apoptotic proteins via autophagy. Autophagy can promote apoptosis by degrading inhibitors of apoptosis proteins (IAPs), including c-FLIP and Mcl-1, which impede apoptotic signals [60]. The depletion of these proteins may render cells more susceptible to apoptosis, hence increasing cell death in response to chemotherapeutic drugs. Conversely, apoptosis can inhibit autophagy by cleaving critical autophagy-related proteins. Caspases, particularly caspase-3 and caspase-8, have been shown to cleave Beclin-1, thereby inhibiting autophagy and directing the cell towards apoptosis [16]. This reciprocal regulation suggests that, whereas autophagy may delay apoptosis under specific conditions, excessive or prolonged autophagy may ultimately lead to apoptosis-dependent cell death [61]. In cancer treatment, the induction of autophagy alongside the targeting of apoptotic pathways has surfaced as a promising technique to augment tumor cell mortality [62]. Chemotherapeutic agents such as doxorubicin and cisplatin have been shown to induce autophagy, which, when combined with apoptosis-inducing medications, leads to increased cancer cell mortality [63]. Nonetheless, the result is significantly contingent on context, as autophagy may function as a survival strategy by destroying pro-apoptotic proteins.

#### 2.3.2. Autophagy and Necroptosis: A Complex Interplay

Necroptosis is a type of controlled necrosis facilitated by receptor-interacting protein kinases (RIPK1 and RIPK3) and the mixed lineage kinase domain-like protein (MLKL) [64]. In contrast to apoptosis, necroptosis does not engage caspase activation and is frequently initiated by tumor necrosis factor-alpha (TNF-α) signaling in caspase-deficient environments [65]. Autophagy has been linked to the regulation of necroptosis through various pathways [66]. A key connection between these two processes is the role of autophagy in regulating the activity of RIPK1 and RIPK3. Research indicates that autophagy can selectively destroy RIPK1 through RIPK1ophagy, thereby inhibiting necroptosis and enhancing cell survival [67]. Conversely, the suppression of autophagy may result in the buildup of RIPK1, hence initiating necroptotic cell death. Furthermore, autophagy can modulate MLKL, the ultimate effector of necroptosis [68]. Following necroptotic stimulation, MLKL is phosphorylated and relocates to the plasma membrane, resulting in membrane rupture and cellular demise [69]. Autophagy has been shown to regulate MLKL activation by preferentially degrading phosphorylated MLKL, hence affecting the magnitude of necroptotic signaling [68]. The interplay between autophagy and necroptosis is particularly significant in cancer, as numerous tumor cells develop resistance to apoptosis due to alterations in key apoptotic genes [70]. In these instances, generating necroptosis via autophagy suppression may offer an alternate approach to elicit tumor cell death. Numerous studies have demonstrated that targeting autophagy-related proteins, such as ATG5 or Beclin-1, can increase the susceptibility of cancer cells to necroptotic death, highlighting the therapeutic potential of this interaction.

#### 2.3.3. Autophagy and Ferroptosis: The Role of Ferritinophagy

Ferroptosis is a unique type of iron-dependent cellular demise characterized by the accumulation of lipid peroxides and oxidative stress [71]. In contrast to apoptosis and necroptosis, ferroptosis is predominantly governed by iron metabolism, lipid peroxidation, and antioxidant defense systems [72]. Ferritinophagy, a selective autophagic process that degrades ferritin, the iron-storage protein, provides a crucial connection between autophagy and ferroptosis [73]. Ferritinophagy is facilitated by nuclear receptor coactivator 4 (NCOA4), which promotes the autophagic breakdown of ferritin, resulting in the liberation of free iron [74]. The elevation of free iron concentrations amplifies the generation of reactive oxygen species (ROS) via the Fenton reaction, consequently facilitating lipid peroxidation and ferroptotic cell death [75]. The interaction between autophagy and ferroptosis in cancer has garnered interest as a potential treatment strategy [76]. Specific tumor cells demonstrate resistance to ferroptosis by enhancing antioxidant mechanisms, including glutathione peroxidase 4 (GPX4), to counteract lipid peroxides [76]. Nonetheless, the modification of autophagy can disrupt this equilibrium by enhancing ferritinophagy, consequently elevating iron availability and rendering cells more susceptible to ferroptosis [74]. Furthermore, specific autophagy inhibitors, such as chloroquine and bafilomycin A1, have demonstrated the capacity to block ferritinophagy, thereby reducing free iron concentrations and preventing ferroptosis [77]. In contrast, ferroptosis inducers such as erastin and RSL3 can promote autophagy-mediated degradation of ferritin, thereby intensifying ferroptotic cell death [78]. This suggests that targeting ferritinophagy in conjunction with ferroptosis inducers may be a practical approach to eliminate cancer cells.

## 3. Role of Autophagy-Dependent Cell Death in Cancer Therapy

### 3.1. Dual Role of Autophagy-Dependent Cell Death in Cancer

**Figure 4:**
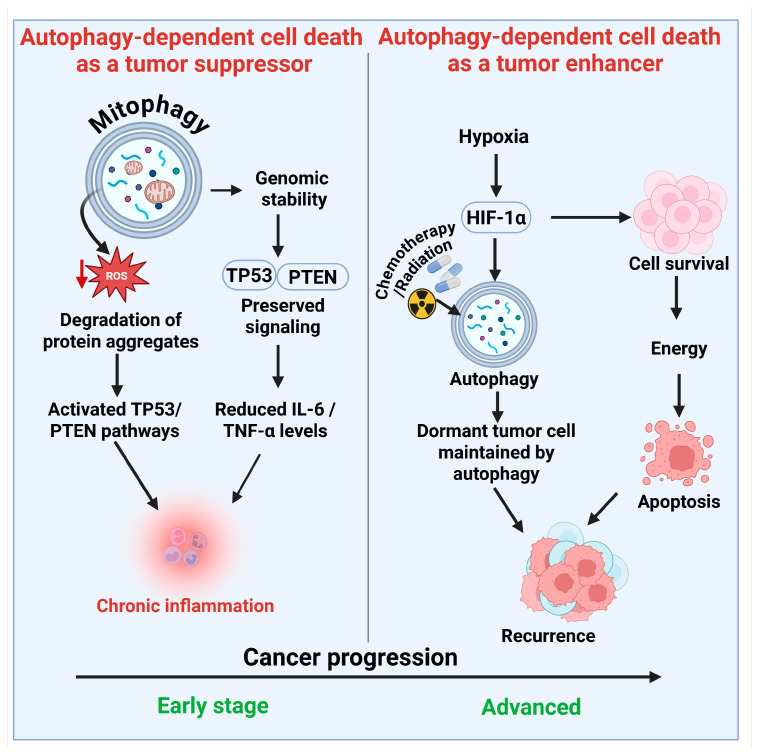
The dual role of autophagy-dependent cell death in cancer progression and therapy resistance. (**Left Panel**) (Tumor Suppressor Role): In the initial stages of cancer development, autophagy facilitates the removal of damaged organelles through mitophagy, decreases reactive oxygen species (ROS), and preserves genomic stability. It promotes the degradation of misfolded protein aggregates, activates tumor suppressor pathways (TP53 and PTEN), and suppresses chronic inflammation by reducing pro-inflammatory cytokines (IL-6, TNF-α), thereby inhibiting malignant transformation. (**Right Panel**) The right panel illustrates the role of autophagy as a tumor enhancer in advanced cancer, facilitating tumor cell survival during stress conditions such as hypoxia and nutrient deprivation, primarily through HIF-1α signaling. It improves resistance to chemotherapy and radiation by maintaining cellular integrity and inhibiting apoptosis. Autophagy sustains quiescent tumor cells in a dormant state, thereby facilitating recurrence after treatment. The shift in autophagy from a suppressive to a promotive role takes place throughout the cancer progression timeline, underscoring its complexity as a therapeutic target. The figure was modified and created using the BioRender.com online commercial platform.

Autophagy-dependent cell death (ADCD) plays a dual role in cancer therapy, acting both as a tumor suppressor and a cell survival mechanism. It can either promote or inhibit cancer cell death, depending on the context and specific cellular conditions (Figure 4).

#### 3.1.1. Autophagy-Dependent Cell Death Functions as a Tumor Suppressor

Autophagy-dependent cell death may function as a tumor suppressor. Although autophagy may facilitate the survival of cancer cells, its role in removing damaged cells and maintaining genomic stability during the initial phases of carcinogenesis is crucial for preventing tumor progression [79].

##### Preservation of Cellular Homeostasis and Genomic Integrity

Autophagy serves as a quality control system, mitigating cellular stress that can lead to cancer development. It eliminates malfunctioning mitochondria by mitophagy, hence decreasing excessive ROS generation, which is recognized to induce DNA damage and genetic alterations [80]. The removal of impaired organelles reduces the likelihood of cellular transformation and tumorigenesis.

##### Regulation of Oncogenes and Tumor Suppressor Genes

Autophagy engages with essential tumor suppressor pathways, including the TP53 (p53) and PTEN signaling pathways, which are vital for inhibiting unregulated cell proliferation [81]. The absence of critical autophagy regulators, such as Beclin-1 (ATG6) and ATG5, has been linked to heightened vulnerability to tumorigenesis in multiple malignancies, including breast and ovarian cancers [82]. Furthermore, autophagy inhibits the accumulation of aberrant protein aggregates, which can disrupt cellular signaling pathways and promote oncogenesis. Autophagy serves as a preventive mechanism against malignant transformation by preserving cellular integrity.

##### Mitigation of Chronic Inflammation

Chronic inflammation is a recognized contributor to tumor genesis and progression. Dysfunctional autophagy is associated with heightened inflammatory signaling, fostering a pro-tumorigenic microenvironment. The targeted degradation of inflammasomes through autophagy mitigates excessive inflammatory responses, consequently diminishing the probability of tumor initiation [83]. Research indicates that the depletion of ATGs results in heightened release of pro-inflammatory cytokines, including interleukin-6 (IL-6) and tumor necrosis factor-alpha (TNF-α), fostering a milieu favorable for tumorigenesis [84]. Consequently, autophagy helps mitigate carcinogenesis associated with chronic inflammation.

#### 3.1.2. Autophagy-Dependent Cell Death as a Tumor Enhancer

Autophagy inhibits cancer initiation, but in established tumors, it facilitates tumor progression and therapeutic resistance. Cancer cells utilize autophagy to acclimate to adverse environments, such as hypoxia, nutritional scarcity, and chemotherapeutic duress [85].

##### Metabolic Adaptation and Resistance Under Stress

Autophagy primarily facilitates tumor growth by enhancing the survival of cancer cells during metabolic stress [86]. Within the microenvironment of a tumor, cancer cells frequently encounter nutritional scarcity and hypoxia resulting from insufficient blood flow [87]. Autophagy enables these cells to recycle intracellular constituents for energy production and to sustain survival. In hypoxic situations, hypoxia-inducible factor 1-alpha (HIF-1α) stimulates autophagy to supply an alternate energy source [88]. This metabolic adaptability enables cancer cells to survive and proliferate in environments that would otherwise be lethal. Furthermore, autophagy helps tumor cells mitigate oxidative stress by removing damaged mitochondria and reducing elevated levels of ROS [89]. This protective process enhances tumor cell viability and promotes resistance to various therapies.

##### Role in Therapy Resistance

Autophagy plays a crucial role in facilitating resistance to chemotherapy and radiation therapy. Numerous anticancer therapies function by eliciting cellular stress and facilitating apoptotic cell death [90]. Cancer cells can induce autophagy as a survival strategy, mitigating the damaging effects of these treatments. Cancer cells subjected to chemotherapy drugs including cisplatin, doxorubicin, and temozolomide, frequently demonstrate heightened autophagy activity [91]. This facilitates the degradation of impaired organelles and sustains cellular activity, hence diminishing the efficacy of chemotherapy. Radiation therapy causes DNA damage and oxidative stress in neoplastic cells [92]. Autophagy alleviates this damage by eliminating impaired organelles and facilitating cellular repair, hence enhancing tumor cells' resistance to radiation-induced apoptosis [93]. In tumors subjected to targeted therapy, such as tyrosine kinase inhibitors (e.g., erlotinib, imatinib), autophagy is frequently elevated as a compensatory mechanism to maintain tumor cell viability [94]. Autophagy inhibition has been proposed as a method to enhance the efficacy of targeted medications.

##### Role in Tumor Dormancy and Recurrence

Autophagy contributes to tumor dormancy and recurrence [95]. Specific cancer cells enter a latent state, characterized by a quiescent phase with diminished metabolic activity. This enables them to circumvent immune surveillance and withstand conventional therapy. Autophagy sustains these quiescent cells by preserving modest energy demands and inhibiting apoptosis. After therapy, dormant cells may reawaken and lead to the recurrence of cancers. This highlights the need for therapeutic approaches that focus on autophagy to prevent tumor recurrence.

### 3.2. Therapeutic Targeting of Autophagy in Cancer

Due to its dual nature, researchers have explored various techniques to regulate autophagy for therapeutic purposes. Inhibiting autophagy may enhance the efficacy of specific cancer therapies, while activating ADCD can also serve as a method to eliminate cancer cells. This section examines the principal strategies for modulating autophagy in cancer treatment, encompassing both the suppression and activation of autophagy.

#### 3.2.1. Autophagy Inhibition in Cancer Therapy

##### Chloroquine (CQ) and Hydroxychloroquine (HCQ): Inhibiting Autophagosome-Lysosome Fusion

Chloroquine (CQ) and its derivative, hydroxychloroquine (HCQ), are extensively researched autophagy inhibitors that operate by obstructing the fusion of autophagosomes with lysosomes [96]. These pharmaceuticals elevate the pH of lysosomes, thereby hindering their degradative capabilities and leading to the accumulation of defective organelles and misfolded proteins [97]. CQ and HCQ have been examined in numerous preclinical and clinical trials for their potential to augment the effectiveness of standard cancer treatments [98]. In glioblastoma, pancreatic cancer, and melanoma, CQ/HCQ has demonstrated the ability to enhance chemotherapy efficacy by inhibiting cancer cells from utilizing autophagy as a survival strategy [99]. CQ has been shown to inhibit tumor angiogenesis and metastasis, positioning it as a promising candidate for combination therapy [100]. The efficacy of CQ and HCQ varies among different cancer types, necessitating further research to enhance their therapeutic applications. CQ and its analog hydroxychloroquine, inhibit lysosomal acidification, blocking autophagic flux and causing autophagosome accumulation. While often cytoprotective, in some contexts, this overload triggers ADCD or apoptosis. In liposarcoma cells, CQ, paired with rapamycin, caused extensive apoptosis via increased autophagosomes via the ADCD pathway [100].

##### mTOR Inhibitors: Inhibiting Autophagy Activation in Cancers

**Figure 5:**
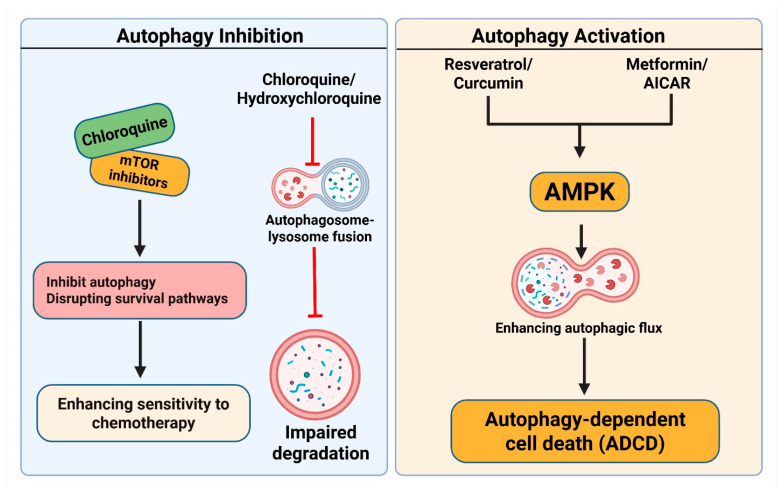
Pharmacological modulation of Autophagy-dependent cell death in cancer therapy. (**Left panel**): Autophagy Inhibition: Chloroquine (CQ) and hydroxychloroquine (HCQ) inhibit the fusion of autophagosomes and lysosomes by increasing lysosomal pH, resulting in compromised degradation of cellular components. mTOR inhibitors have been demonstrated to indirectly inhibit autophagy or act in conjunction with CQ, thereby disrupting survival pathways and enhancing sensitivity to chemotherapy. (**Right panel**): Autophagy Activation: Resveratrol, curcumin, metformin, and AICAR stimulate the AMPK signaling pathway, enhancing autophagic flux. This results in ADCD, which is especially advantageous in apoptosis-resistant cancer cells. The activation of AMPK through metabolic stress or pharmacological agents facilitates the formation of autophagosomes and the degradation of oncogenic proteins. The figure was modified and created using the BioRender.com online commercial platform.

The mechanistic target of rapamycin (mTOR) serves as a primary regulator of autophagy [101]. The activation of mTOR inhibits autophagy, while the inhibition of mTOR increases it [102]. In certain tumors, paradoxically, overactive mTOR signaling inhibits autophagy and facilitates tumor proliferation [103]. In certain instances, cancer cells depend on autophagy for survival in stressful environments. mTOR inhibitors, including rapamycin and its analogs (temsirolimus and everolimus), have been studied for their ability to inhibit autophagy-mediated tumor growth [104]. mTOR inhibitors typically serve as autophagy inducers, although they may also work as indirect inhibitors when used in conjunction with other autophagy-inhibiting drugs. For instance, the concurrent suppression of mTOR and autophagy with a combination of rapamycin and CQ has demonstrated potential in preclinical investigations [105]. This method efficiently inhibits cancer cells from transitioning to an autophagy-dependent survival mechanism after mTOR inhibition (Figure 5).

#### 3.2.2. Activation of Autophagy in Cancer Treatment

Although autophagy inhibition is advantageous in particular cancer types, the activation of autophagy may also serve as a practical approach to cause ADCD. This method is especially beneficial in apoptosis-resistant tumors, where autophagy may function as an alternate mechanism of cell killing. A variety of natural substances and pharmaceutical treatments have been examined for their capacity to enhance ADCD in cancer cells.

##### Resveratrol and Curcumin: Facilitating ADCD to Augment Apoptosis in Cancer Cells

Natural substances, such as resveratrol and curcumin, have been extensively researched for their anticancer properties, particularly their ability to regulate autophagy [106]. A polyphenolic compound present in grapes and red wine, resveratrol stimulates autophagy by blocking mTOR and activating the AMP-activated protein kinase (AMPK) pathway [107]. It has been demonstrated to elicit ADCD in multiple cancer cell lines, including those of breast, prostate, and colon cancers. Resveratrol additionally amplifies the cytotoxic effects of chemotherapy by facilitating autophagy-mediated degradation of anti-apoptotic proteins [108]. Extracted from turmeric, curcumin is a potent autophagy inducer with many anticancer properties [109]. It facilitates ADCD by augmenting autophagic flux and obstructing critical survival pathways in neoplastic cells. Research indicates that curcumin enhances the sensitivity of cancer cells to chemotherapy and radiotherapy by stimulating the ADCD pathway, while diminishing inflammation and oxidative stress [110]. Additionally, curcumin can induce non-apoptotic/autophagy-dependent death. In renal carcinoma, co-treatment with PP242 (mTORC1/2 inhibitor) and curcumin caused apoptosis and ADCD via lysosomal damage, which was suppressed by autophagy inhibitors [111]. Resveratrol and curcumin have been evaluated in combination with conventional medicines to enhance their anticancer effectiveness [112]. Nonetheless, their bioavailability poses a challenge, necessitating further research to improve their therapeutic applicability.

##### AMPK Activators: Augmenting Autophagic Flux to Induce Cytotoxicity in Cancer

AMPK is a crucial energy sensor that modulates cellular metabolism and autophagy. Activation of AMPK enhances autophagic flux and causes ADCD, positioning it as a potential target for cancer treatment [113]. A variety of pharmacological substances and metabolic stresses can stimulate AMPK activation [114]. A commonly utilized antidiabetic medication, metformin stimulates AMPK and promotes autophagy in neoplastic cells [115]. Preclinical chloroquine studies suggest that metformin-induced autophagy activation may enhance ADCD in certain cancer types, particularly those with metabolic vulnerabilities [116]. In H4IIE cells under glucose deprivation, metformin suppressed autophagy and triggered apoptosis through AMPK pathways by ADCD. In osteosarcoma, metformin was linked to reduced metastasis by affecting autophagy-related mechanisms in ADCD [117]. As a direct activator of AMPK, AICAR (5-Aminoimidazole-4-carboxamide ribonucleotide) has demonstrated the ability to induce autophagy and inhibit tumor growth in experimental animals [118]. AICAR increases cancer cell death by augmenting the autophagic breakdown of oncogenic proteins [119]. Lifestyle modifications, including exercise and intermittent fasting, have been shown to activate AMPK and autophagy, thereby enhancing their potential anticancer properties [120]. Although these techniques are not independent medicines, they may improve current treatment strategies by altering tumor metabolism.

### 3.3. Combination Strategies for Enhancing Cancer Therapy

**Figure 6:**
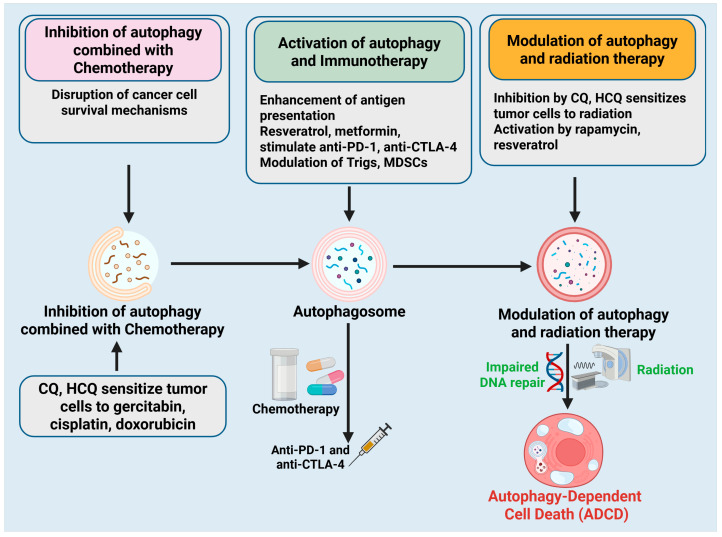
Combination strategies using Autophagy-dependent cell death modulation in cancer therapy. Three principal combinatorial techniques that employ autophagy manipulation to improve cancer treatment efficacy. (**Left panel**)**:** Autophagy inhibition in conjunction with chemotherapy. Treatments like CQ and HCQ interfere with autophagy-dependent survival mechanisms in cancer cells, rendering them more susceptible to chemotherapeutic treatments such as gemcitabine, cisplatin, and doxorubicin. (**Middle panel**)**:** The activation of autophagy augments immunotherapy. Autophagy inducers (e.g., resveratrol, metformin) enhance antigen presentation and immune cell activation, hence augmenting the efficacy of immune checkpoint inhibitors such as anti-PD-1 and anti-CTLA-4. Autophagy reprograms immunosuppressive cells, such as Tregs and MDSCs, into pro-inflammatory phenotypes. (**Right panel**)**:** Regulation of autophagy in conjunction with radiation therapy. CQ and HCQ augment radiosensitivity by disrupting DNA repair processes, whereas compounds such as rapamycin and resveratrol may facilitate ADCD, hence amplifying the harmful effects of radiation. The figure was modified and created using the BioRender.com online commercial platform.

This complexity has prompted the investigation of combinatorial techniques that incorporate autophagy modulation with standard cancer therapies, including chemotherapy, immunotherapy, and radiation therapy. Researchers seek to enhance therapeutic efficacy and defeat drug resistance by modulating autophagy, either through inhibition or activation, in a context-dependent manner. This section examines essential combinatorial strategies that utilize autophagy modulation to improve cancer therapy (Figure 6).

#### 3.3.1. Inhibition of Autophagy Combined with Chemotherapy: Disrupting Mechanisms of Cancer Cell Survival

Chemotherapy is a fundamental component of cancer treatment; its efficacy is frequently constrained by drug resistance. Inhibiting autophagy has become a viable strategy to augment chemotherapy effectiveness by obstructing cancer cells from utilizing autophagy as a survival mechanism [121]. Numerous chemotherapeutic drugs elicit metabolic stress and DNA damage, activating protective autophagy in tumor cells [122]. This reaction enables cancer cells to evade apoptosis, resulting in therapy failure. Inhibitors like CQ and HCQ impede autophagy, hence undermining this survival mechanism and rendering cancer cells more vulnerable to chemotherapy-induced apoptosis [123]. In pancreatic cancer, CQ has demonstrated the ability to augment the effectiveness of gemcitabine, a conventional chemotherapy agent [124]. In breast and lung malignancies, the suppression of autophagy enhances the cytotoxic efficacy of cisplatin and doxorubicin [125]. These findings support the use of autophagy inhibitors in combination with chemotherapy to mitigate drug resistance and improve patient outcomes.

#### 3.3.2. Activation of Autophagy and Immunotherapy: Augmenting Immune-Mediated Tumor Elimination

Immunotherapy has transformed cancer treatment by utilizing the body's immune system to identify and eliminate cancerous cells [126]. Nonetheless, immune evasion continues to pose a considerable obstacle, hindering the efficacy of checkpoint inhibitors, adoptive T-cell therapy, and cancer vaccines. Recent studies suggest that activating autophagy can enhance the efficacy of immunotherapy by facilitating antigen presentation and stimulating immune cell activation [127]. Autophagy improves tumor detection by cytotoxic T lymphocytes by augmenting the degradation of tumor-associated antigens and their presentation on major histocompatibility complex (MHC) molecules [128]. Furthermore, autophagy modulates the function of immune cells, encompassing the viability and activity of dendritic cells, macrophages, and natural killer (NK) cells [129]. Autophagy inducers, such as resveratrol and metformin, have demonstrated the ability to enhance the efficacy of immune checkpoint inhibitors (e.g., anti-PD-1, anti-CTLA-4) in models of melanoma and lung cancer [130]. Moreover, in solid tumors characterized by an immunosuppressive microenvironment, the activation of autophagy can facilitate the reprogramming of immune-suppressive cells, including regulatory T cells (Tregs) and myeloid-derived suppressor cells (MDSCs), into pro-inflammatory phenotypes [131]. This method increases immune-mediated tumor eradication and amplifies the enduring advantages of immunotherapy.

#### 3.3.3. Modulation of Autophagy and Radiation Therapy: Enhancing Therapeutic Efficacy in Resistant Tumors

Radiation therapy is a prevalent treatment for several cancers, yet radioresistance continues to be a significant challenge. Modulating autophagy has emerged to increase the sensitivity of tumor cells to radiation. Radiation causes DNA damage and oxidative stress, prompting the activation of autophagy as a defensive mechanism. Inhibition of autophagy by CQ or HCQ has been demonstrated to augment radiosensitivity in glioblastoma, prostate cancer, and cervical cancer by obstructing DNA repair and promoting tumor cell mortality [132]. Conversely, in certain instances, the activation of autophagy may enhance radiation efficacy by facilitating ADCD. Autophagy inducers, such as rapamycin and resveratrol, have demonstrated an enhancement of radiation-induced cytotoxicity in particular cancer types, including colorectal and lung malignancies [133]. The context-specific function of autophagy in radiation therapy highlights the need for personalized treatment strategies based on tumor classification and autophagy status.

## 4. Therapeutic and Clinical Application of Autophagy-Dependent Cell Death in Cancer Therapy

As our knowledge of ADCD advances, researchers and clinicians are intensively investigating its practical uses to enhance patient outcomes. ADCD is essential in regulating cellular survival and apoptosis, rendering it a significant target for therapeutic strategies. In contrast to apoptosis, which genetic changes can circumvent, ADCD offers an alternate strategy for tumor eradication. Therapies aimed at activating ADCD can provoke autophagic stress in cancer cells, resulting in their self-destruction. Table 1 outlines several autophagy-modulating pharmaceuticals with therapeutic potential in cancer treatment, either by activating or suppressing autophagy to facilitate ADCD. These pharmaceuticals are being investigated in preclinical and clinical settings to enhance cancer treatment efficacy. In cancers where autophagy facilitates survival and therapeutic resistance, inhibiting autophagy is a feasible approach to improve treatment effectiveness. Conversely, stimulating autophagy may serve as an advantageous approach in tumors where autophagy-mediated cell death facilitates tumor reduction. Various substances efficiently induce ADCD via distinct pathways.

Chloroquine (CQ) and Hydroxychloroquine (HCQ) are frequently utilized for the treatment of malaria and autoimmune diseases, but they have also been repurposed for cancer treatments. They operate by obstructing the union of autophagosomes and lysosomes, therefore averting the breakdown of impaired proteins and organelles. CQ and HCQ have demonstrated potential in glioblastoma, breast, pancreatic, and lung malignancies by augmenting sensitivity to chemotherapy and radiation [134,135]. Bafilomycin A1 is a potent lysosomal inhibitor that obstructs lysosomal acidification, thereby hindering autophagic flow. It has demonstrated efficacy in liver cancer and leukemia, wherein the inhibition of autophagy results in the accumulation of toxic waste within cancer cells, ultimately leading to cell death [136]. 3-Methyladenine (3-MA)-A is a recognized PI3K inhibitor. 3-MA obstructs the onset of autophagy, rendering it a valuable approach in the treatment of colorectal and lung cancer. 3-MA amplifies the cytotoxic effects of chemotherapeutic drugs by diminishing autophagic activity [137]. Rapamycin, Everolimus, and Temsirolimus are mTOR inhibitors that induce autophagy by inhibiting mTORC1, a key regulator of cellular growth and survival. Rapamycin has shown effectiveness in renal cell carcinoma and breast cancer [138], but everolimus is sanctioned for the treatment of neuroendocrine tumors [139]. Temsirolimus has been investigated in lymphomas, where it increases tumor susceptibility to chemotherapeutic therapies [140]. Resveratrol and Curcumin, being natural substances, facilitate autophagy by causing oxidative stress and activating Beclin-1, a crucial regulator of autophagy. Resveratrol has demonstrated efficacy in colon cancer and melanoma [141], whilst curcumin has exhibited potential in lung and pancreatic malignancies [142]. Metformin, widely used for diabetes control, activates AMPK, which in turn inhibits mTOR, thereby facilitating autophagy and ADCD. Research indicates its possible application in prostate and ovarian malignancies, where it amplifies the efficacy of chemotherapy [143]. Sunitinib, a multi-kinase inhibitor, promotes autophagy-dependent cell death via mTORC1 inhibition, rendering it an effective therapy for renal cell carcinoma and gastrointestinal stromal tumors [144]. Doxorubicin is a chemotherapeutic agent. Doxorubicin, a prominent chemotherapeutic agent, promotes autophagy-dependent apoptosis through ROS production, rendering it effective against breast cancer and osteosarcoma [145]. Cisplatin, a prevalent chemotherapy drug, produces autophagic cell death mediated by endoplasmic reticulum stress, rendering it effective against ovarian and bladder malignancies [146]. Vinblastine, a chemotherapeutic agent, interferes with microtubules, resulting in autophagic stress and ADCD, especially in lymphoma and breast cancer [147]. Carbamazepine, initially developed as an anticonvulsant, Carbamazepine has demonstrated the ability to augment autophagic flux and the breakdown of damaged proteins, positioning it as a possible treatment candidate for glioblastoma and pancreatic cancer [148].

## 5. Limitations and Future Directions of Autophagy-Dependent Cell Death in Cancer Therapy

Although ADCD holds promise in cancer treatment, several obstacles and limitations must be addressed to enhance its clinical implementation. By overcoming these challenges, autophagy-targeted therapies have the potential to transform cancer treatment, thereby improving patient outcomes and reducing therapeutic resistance. During early carcinogenesis, autophagy mitigates genomic instability, whereas in advanced malignancies, it facilitates survival in the face of metabolic stress. Cancer cells within a singular tumor demonstrate varied responses to autophagy regulation. Specific cells may experience ADCD, whereas others may acquire resistance, resulting in variable treatment results. Numerous autophagy-targeting pharmaceuticals, including CQ, HCQ, and rapamycin, have off-target effects that disrupt normal cellular homeostasis and induce detrimental side effects [5]. Extended regulation of autophagy may induce adaptive resistance in cancer cells, diminishing therapeutic effectiveness over time. This requires the formulation of combination medicines to maintain enduring advantages. Identifying precise biomarkers for autophagy activity in cancers will facilitate the development of targeted treatment techniques, thereby enhancing drug selection for patients. The integration of autophagy modulators with chemotherapy, immunotherapy, or radiation therapy may augment efficacy and surmount drug resistance [125]. For instance, the suppression of autophagy might augment chemotherapy-induced apoptosis, whilst the activation of autophagy can promote immunological responses [15]. Future research should concentrate on the development of targeted autophagy modulators that reduce off-target effects and specifically target neoplastic tissues. Translational research and the addition of clinical trials are required to evaluate the long-term advantages and safety of autophagy-based therapy for various types of cancer.

Defining and displaying Autophagy-Dependent Cell Death (ADCD) in cancer models is challenging because of its dual role in determining cell fate. Autophagy frequently fulfills a cytoprotective role, allowing cancer cells to deal with stressors such as chemotherapy and hypoxia. Determining ADCD requires unequivocal evidence that the autophagy machinery is not only active but also crucial for the execution of cell death, independent of apoptosis or necrosis. This distinction is contentious, as many studies indicate that autophagy enhances survival rather than induces mortality. Furthermore, pharmacological and genetic instruments exhibit a lack of selectivity, complicating the differentiation between autophagy-dependent effects and off-target toxicity or interactions with alternative cell death pathways.

## 6. Conclusions

Autophagy-Dependent Cell Death (ADCD) is a significant and complex domain in cancer treatment, providing the possibility of eradicating tumor cells by the regulated initiation of self-digestive processes. In contrast to conventional apoptosis-targeted therapies, ADCD utilizes a fundamentally different cellular pathway, providing an alternate method for eradicating resistant cancer morphologies. Nonetheless, its utilization is constrained by an inadequate comprehension of the conditions under which autophagy transitions from a survival process to a fatal one. A significant barrier exists in accurately differentiating ADCD from cytoprotective autophagy and other modalities of cell death, both in terms of mechanisms and experimental approaches. Furthermore, the influence of tumor-specific contexts, such as microenvironmental stress, genetic alterations, and metabolic state-introduces further complexity to the targeting of autophagy for therapeutic benefit. Going forward, a comprehensive strategy that integrates molecular profiling, real-time autophagic flux monitoring, and targeted genetic or pharmacological manipulation will be essential. Identifying predictive biomarkers that indicate the functional involvement of autophagy in a certain tumor could facilitate patient classification and customized treatment. Ultimately, although ADCD presents potential as a revolutionary approach in oncology, its clinical use relies on a comprehensive molecular understanding, context-specific validation, and meticulous therapy design to minimize off-target effects and enhance efficacy. Subsequent studies must address these deficiencies to realize their medicinal potential fully.

## Figures and Tables

**Table 1:** Pharmaceutical agents that modulate ADCD, their methods of action, and their clinical applications in cancer treatment.

Drug Name	Cancer Type	Mechanism of Action	ADCD	Ref.
Chloroquine (CQ)	Glioblastoma, Breast Cancer	Inhibits autophagosome-lysosome fusion, preventing degradation of cellular components	Inhibition	[134]
Hydroxychloroquine (HCQ)	Pancreatic Cancer, Lung Cancer	Blocks lysosomal acidification, disrupting autophagic flux	Inhibition	[135]
Bafilomycin A1	Liver Cancer, Leukemia	Inhibits lysosomal acidification, blocking autophagosome clearance	Inhibition	[136]
3-Methyladenine (3-MA)	Colorectal Cancer, Lung Cancer	Blocks PI3K-mediated autophagy initiation	Inhibition	[137]
Rapamycin	Renal Cell Carcinoma, Breast Cancer	mTORC1 inhibitor, induces autophagy leading to ADCD	Activation	[138]
Everolimus	Neuroendocrine Tumors, Breast Cancer	mTORC1 inhibitor, enhances autophagic cell death in tumors	Activation	[139]
Temsirolimus	Renal Cell Carcinoma, Lymphoma	mTORC1 inhibition promotes sustained autophagy and tumor regression	Activation	[140]
Resveratrol	Colon Cancer, Melanoma	Induces oxidative stress and autophagic cell death via AMPK activation	Activation	[141]
Curcumin	Lung Cancer, Pancreatic Cancer	Triggers autophagy through Beclin-1 upregulation, enhancing ADCD	Activation	[142]
Metformin	Prostate Cancer, Ovarian Cancer	Activates AMPK, inhibits mTOR signaling, promoting autophagic cell death	Activation	[143]
Sunitinib	Renal Cell Carcinoma, Gastrointestinal Stromal Tumors	Induces autophagy-dependent cell death through mTORC1 inhibition	Activation	[144]
Doxorubicin	Breast Cancer, Osteosarcoma	Enhances autophagy-dependent apoptosis via ROS generation	Activation	[145]
Cisplatin	Ovarian Cancer, Bladder Cancer	Induces ER stress-mediated autophagic cell death	Activation	[146]
Vinblastine	Lymphoma, Breast Cancer	Disrupt microtubules, triggering autophagic stress and ADCD	Activation	[147]
Carbamazepine	Glioblastoma, Pancreatic Cancer	Enhances autophagic flux and degradation of damaged proteins	Activation	[148]

## Data Availability

No new data were created or analyzed in this study.
